# Association between diagnostic efficacy of acoustic radiation force impulse for benign and malignant thyroid nodules and the presence or absence of non-papillary thyroid cancer: A meta-analysis

**DOI:** 10.3389/fonc.2023.1007464

**Published:** 2023-01-27

**Authors:** Jun Li, Yu-Rui Zhang, Jia-Yu Ren, Qiao-Li Li, Pei-Shan Zhu, Ting-Ting Du, Xiao-Yan Ge, Ming Chen, Xin Wu Cui

**Affiliations:** ^1^ Department of Ultrasound, The First Affiliated Hospital of Medical College, Shihezi University, Shihezi, China; ^2^ NHC Key Laboratory of Prevention and Treatment of Central Asia High Incidence Diseases, First Affiliated Hospital, School of Medicine, Shihezi University, Shihezi, China; ^3^ Department of Medical Ultrasound, Tongji Hospital, Tongji Medical College, Huazhong University of Science and Technology, Wuhan, China

**Keywords:** non-papillary thyroid cancer (NPTC), virtual touch quantification (VTQ), virtual touch tissue imaging and quantification (VTIQ), shear wave velocity (SWV), meta-analysis

## Abstract

**Purpose:**

The aim of this study was to investigate the diagnostic efficacy of Acoustic Radiation Force Impulse (ARFI) for benign and malignant thyroid nodules in the presence and absence of non-papillary thyroid cancer (NPTC) and to determine the cut-off values of Shear Wave Velocity (SWV) for the highest diagnostic efficacy of Virtual Touch Quantification (VTQ) and Virtual Touch Tissue Imaging and Quantification (VTIQ).

**Methods:**

The diagnostic accuracy of ARFI for benign and malignant thyroid nodules was assessed by pooling sensitivity, specificity and area under the curve (AUC) in each group in the presence and absence of both non-papillary thyroid glands, using histology and cytology as the gold standard. All included studies were divided into two groups according to VTQ and VTIQ, and each group was ranked according to the magnitude of the SWV cutoff value to determine the SWV cutoff interval with the highest diagnostic efficacy for VTQ and VTIQ.

**Results:**

A total of 57 studies were collected on the evaluation of ARFI for the diagnosis of benign and malignant thyroid nodules. The results showed that the presence of non-papillary thyroid carcinoma led to differences in the specificity of VTIQ for the identification of benign and malignant thyroid nodules, and the differences were statistically significant. In addition, the diagnostic efficacy of VTQ was best when the cutoff value of SWV was in the interval of 2.48-2.55 m/s, and the diagnostic efficacy of VTIQ was best when the cutoff value of SWV was in the interval of 3.01-3.15 m/s.

**Conclusion:**

VTQ and VTIQ have a high diagnostic value for benign and malignant thyroid nodules; however, when the malignant nodules in the study contain non-papillary thyroid carcinoma occupying the thyroid gland, the findings should be viewed in a comprehensive manner.

## Introduction

Thyroid nodules are a very common thyroid disorder and the incidence of thyroid nodules has shown an increasing trend year by year over the last few decades (1). Thyroid cancer accounts for 5% of thyroid nodules (2). There are four main types of thyroid cancer pathology: papillary, follicular, medullary and interstitial. The most common of these pathological types is papillary thyroid cancer (PTC), which also has the best prognosis among thyroid cancers, while the others have a poor prognosis (3). Among them, interstitial thyroid cancer, although less common, is one of the most dangerous tumors and is an associated cause of death in nearly half of thyroid cancer patients (4). Therefore, the first prerequisite for clinical diagnosis is to identify the benign and malignant thyroid nodules and then to develop the most appropriate treatment plan based on this, in order to reduce unnecessary surgeries and surgical complications, and ultimately to improve the quality of life as well as the health status of patients.

Ultrasonography is the test of choice for thyroid disease. Preoperative ultrasound examination of thyroid nodules is the most commonly used clinical method. However, conventional ultrasonography, including color Doppler ultrasound, cannot accurately differentiate between benign and malignant thyroid nodules, even when combined with CT and MRI examinations (1).

Currently, fine-needle aspiration biopsy (FNAB) is one of the recommended adjuncts for the diagnosis of thyroid nodules, but studies have shown that the sensitivity and specificity of FNAB for the diagnosis of thyroid nodules are 65-98% and 72-100%, respectively, and 20%-30% of samples cannot be diagnosed pathologically, with a certain rate of underdiagnosis (5–10). Moreover, FNAB is an invasive procedure with potential complications that have a negative impact on the patient’s health.

In recent years, acoustic radiation force pulse elastography (ARFI) has been widely used in the examination of thyroid diseases, which can reflect the different hardness characteristics of benign and malignant lesions and is very useful for the identification of benign and malignant lesions (11). ARFI includes virtual touch tissue imaging and quantification (VTIQ) and virtual touch tissue quantification (VTQ) techniques, which are based on the principle of measuring the shear wave velocity (SWV) of the regions of interest (ROI) of the tissue. SWV is used to quantify the stiffness of the tissue. In a tissue lesion, the faster the shear wave velocity, the harder the lesion; the slower the shear wave velocity, the softer the lesion (2). Tissue stiffness is a characteristic that can reflect the nature of the nodule. The degree of fibrosis and the number of tumor cells vary among different histologic types of thyroid nodules, resulting in different stiffness in different histologic types of thyroid nodules. Compared to papillary carcinomas, other types of thyroid carcinomas, such as follicular, medullary, and undifferentiated carcinomas exhibit relatively soft structures (12).

In the past, a meta-analysis was performed to evaluate the diagnostic efficacy of ARFI in identifying benign and malignant thyroid nodules, and the results of the study showed that ARFI performed well in the differential diagnosis of benign and malignant thyroid nodules, and that ARFI may help guide the clinical choice of surgery for patients with thyroid nodules (13). However, this study only made a simple benign-malignant distinction between thyroid nodules and did not further delineate the pathological types of thyroid cancer.

The main objective of the present study, taken together with previous studies, was to assess whether the presence of nonpapillary thyroid cancer affects the diagnostic efficacy of ARFI for benign and malignant thyroid nodules and to determine the cut-off interval of SWV with optimal diagnostic efficacy for VTQ and VTIQ.

## Material and methods

### Search strategy

The search databases web of science, PubMed, and Embase were searched for relevant studies published up to May 1, 2022, with the search terms “(Acoustic Radiation Force Impulse or ARFI or VTIQ or VTQ or Virtual Touch tissue imaging and quantification or Virtual Touch tissue quantification) and (thyroid or thyroid nodules)”. The search language was English. In order to search as much relevant literature as possible, the search method of this paper was subject terms combined with free words, web search combined with manual search, and secondary search of the retrieved relevant literature was conducted.

### Study selection

Inclusion criteria: (i) the literature study must include the diagnostic analysis of thyroid nodules by ARFI; (ii) there is a gold standard for diagnosing the pathology of thyroid nodules in the literature, and the number of benign and malignant nodules must be given directly or indirectly; (iii) the number of patients must be ≥30; (iv) the literature should provide raw data and calculate the sensitivity, specificity, false positives and false negatives can be calculated directly or indirectly.

Exclusion criteria: (i) diagnostic criteria were not described; (ii) data could not be extracted; (iii) duplicate literature; (iv) pathological histology was not used as the gold standard; (v) cutoff values for SWV were not indicated; (vi) editorials, letters, case reports, review articles, commentaries, case-control studies, and conference articles.

### Literature inclusion

Two authors independently searched and read the titles, abstracts, and keywords of the detected literature to initially identify eligible literature that could be selected, and then carefully read the full text of the literature to finalize the eligible literature that could be included.

If the 2 authors disagreed on whether the literature should be included, a third author helped to suggest a solution.

### Data extraction

Relevant database literature was screened by 2 independent authors using a blinded method and in strict accordance with the inclusion and exclusion criteria of the literature; those that met the requirements were included and those that did not were excluded. The extracted literature included the authors’ names, the location of the study, the time of publication, the number of included lesions, the number of benign and malignant lesions, the pathology of malignant nodules, and the sensitivity, specificity, and accuracy of the test to be evaluated.

### Statistical analysis

Both stata 16.0 software and RevMan 5.3 software were used for statistical analysis in this study.

The statistical software was used to produce summary receiver operating characteristic (SROC) curves, publication bias funnel plots, and to calculate the sensitivity, specificity, and area under curve (AUC) of the diagnosis, respectively.

### Literature quality evaluation

All included literature was evaluated for quality using RevMan 5.3, a revised tool for quality assessment of diagnostic accuracy studies, including patient selection, index tests, reference standards, processes, and timelines.

## Results

### Literature search results

**Figure 1:**
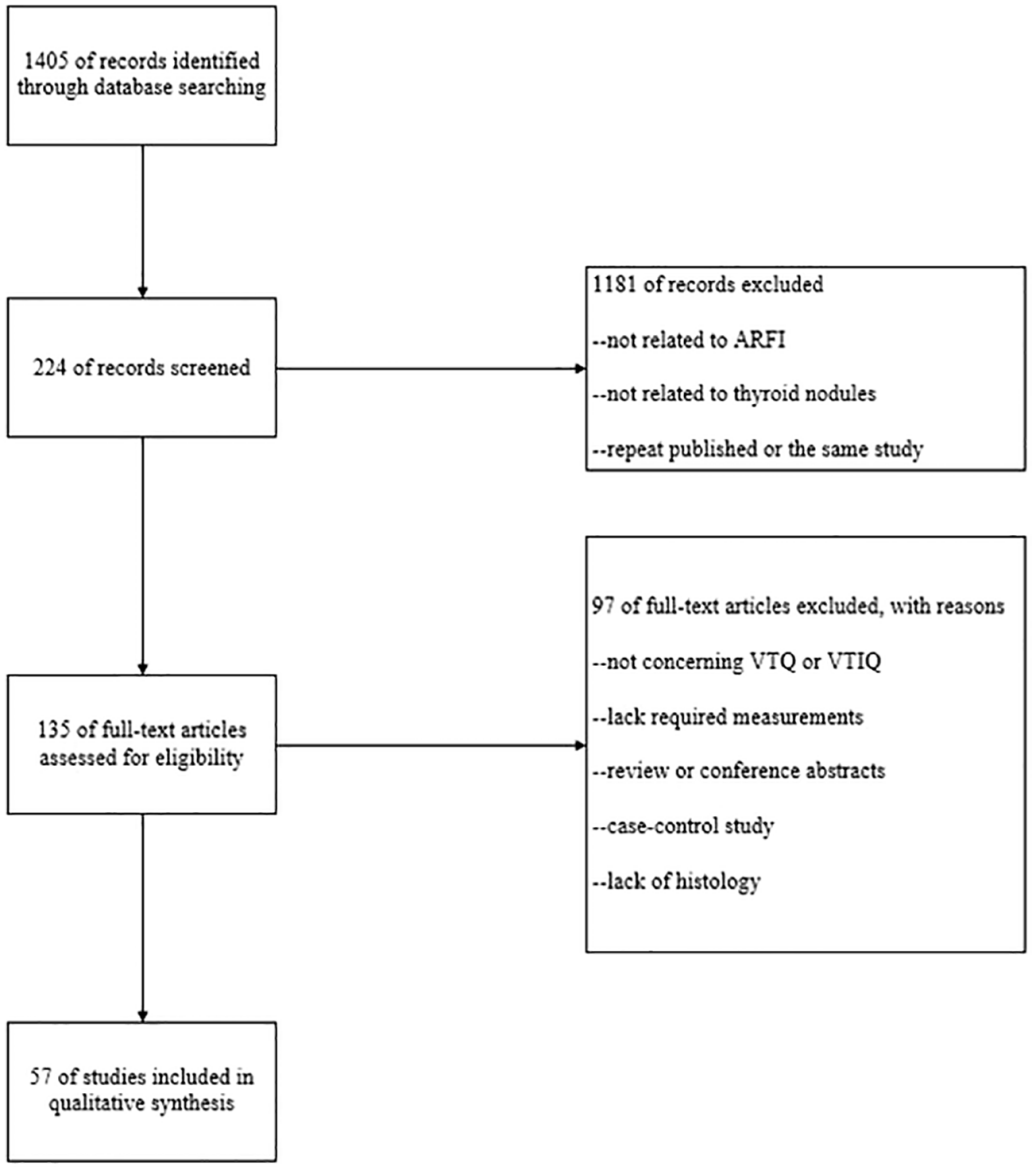
Inclusion and exclusion process of the literature.

In this meta-analysis, 1405 original articles were retrieved based on the search terms “(Acoustic Radiation Force Impulse or ARFI or VTIQ or VTQ or Virtual Touch tissue imaging and quantification or Virtual Touch tissue quantification) and (thyroid or thyroid nodules)”. By carefully reading the titles and abstracts of the articles, 134 papers were initially included, and then the papers were strictly screened and excluded according to the inclusion and exclusion criteria, and finally 57 papers met the conditions of meta-analysis. The specific inclusion process of the literature is shown in **Figure 1**.

### Characteristics of the eligible studies

A total of 8802 thyroid nodules were included in the pooled 57 studies. The nature of all thyroid nodules in all included studies was histologically confirmed. Benign thyroid nodules included nodular goiter, eosinophilia, Hashimoto’s thyroiditis, subacute thyroiditis, and thyroid adenoma, while malignant thyroid nodules included papillary, follicular, undifferentiated, metastatic, and medullary carcinomas.

### Data analysis

**Figure 2:**
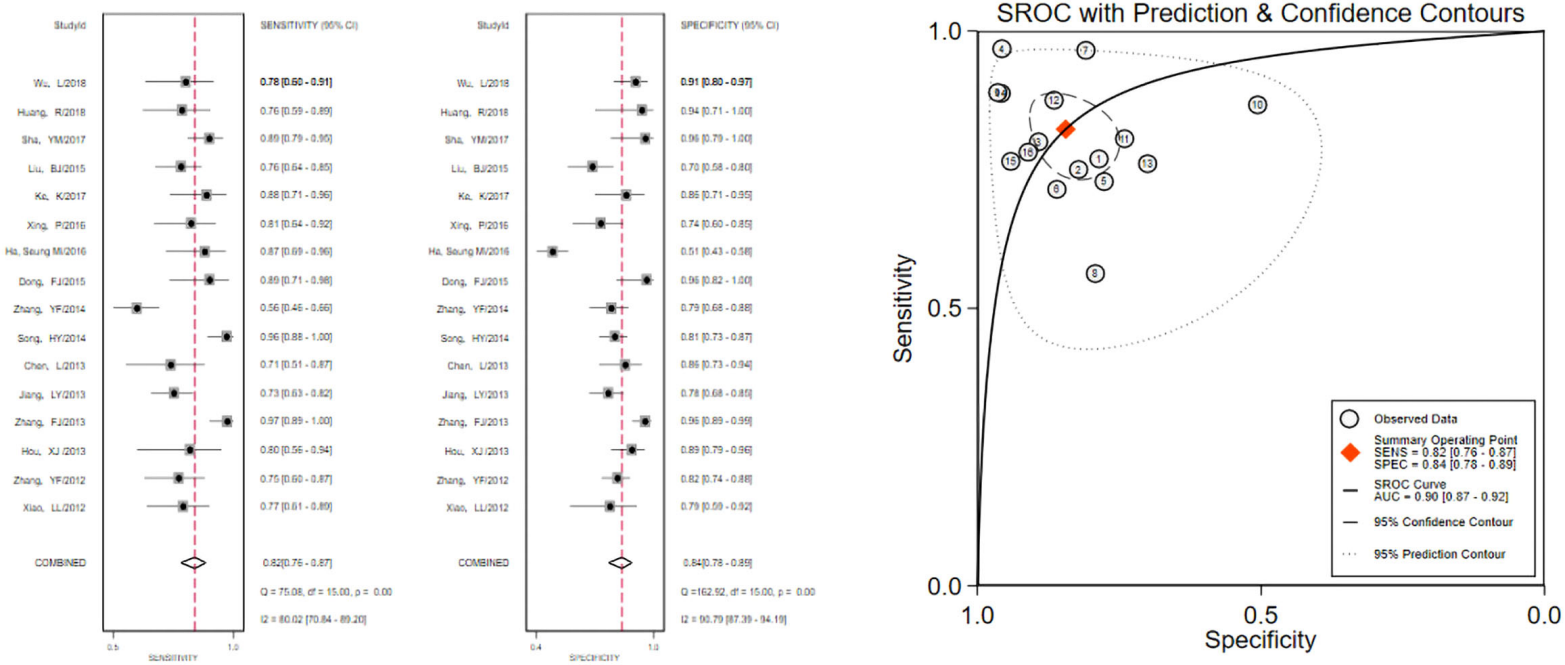
The sensitivity and specificity of the A_VTQ_ group in the diagnosis of thyroid nodules and the summary ROC (Summary ROC) curve of the A_VTQ_ group were analyzed. the AUC indicates the area under the curve.

**Figure 3:**
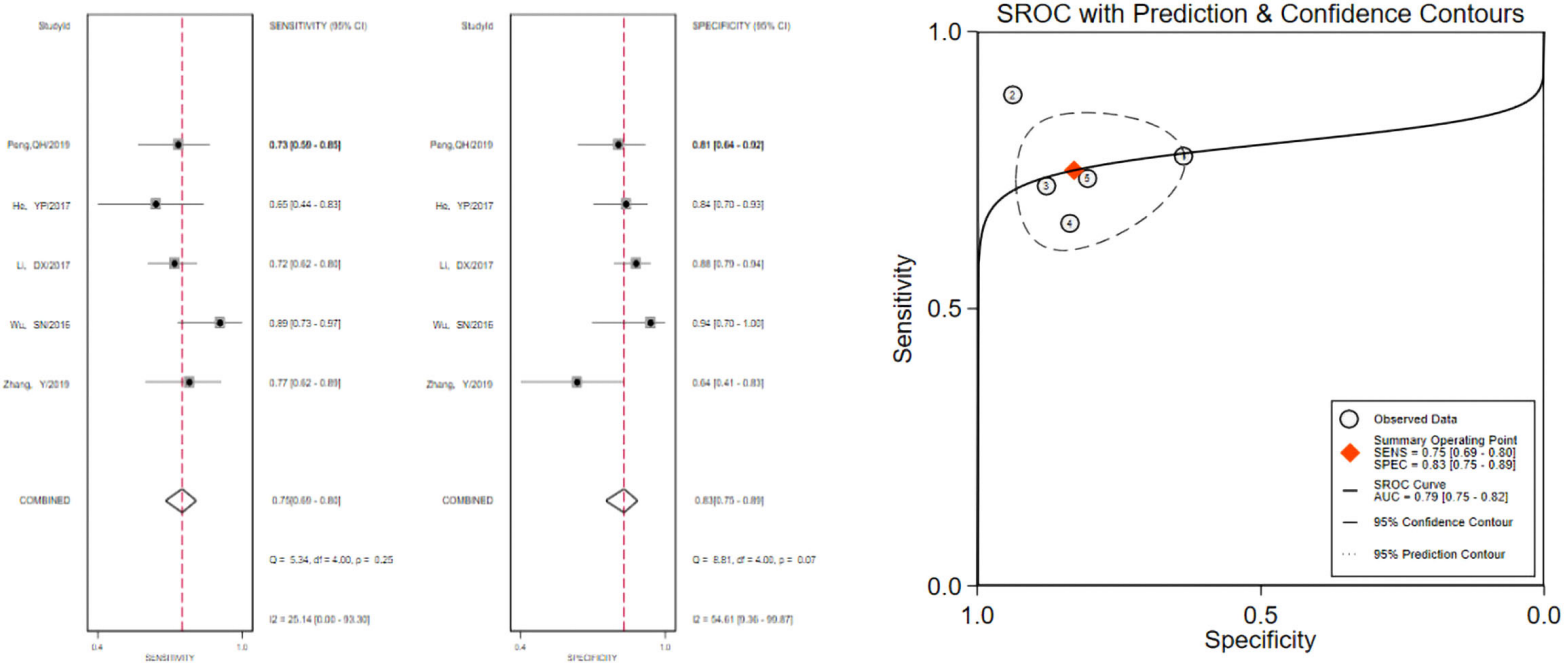
The sensitivity and specificity of the A_VTIQ_ group in the diagnosis of thyroid nodules and the summary ROC (Summary ROC) curve of the A_VTIQ_ group were analyzed. the AUC indicates the area under the curve.

**Figure 4:**
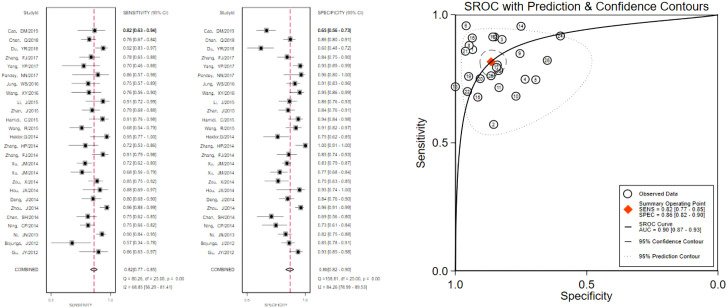
The sensitivity and specificity of the B_VTQ_ group in the diagnosis of thyroid nodules and the summary ROC (Summary ROC) curve of the B_VTQ_ group were analyzed. the AUC indicates the area under the curve.

**Figure 5:**
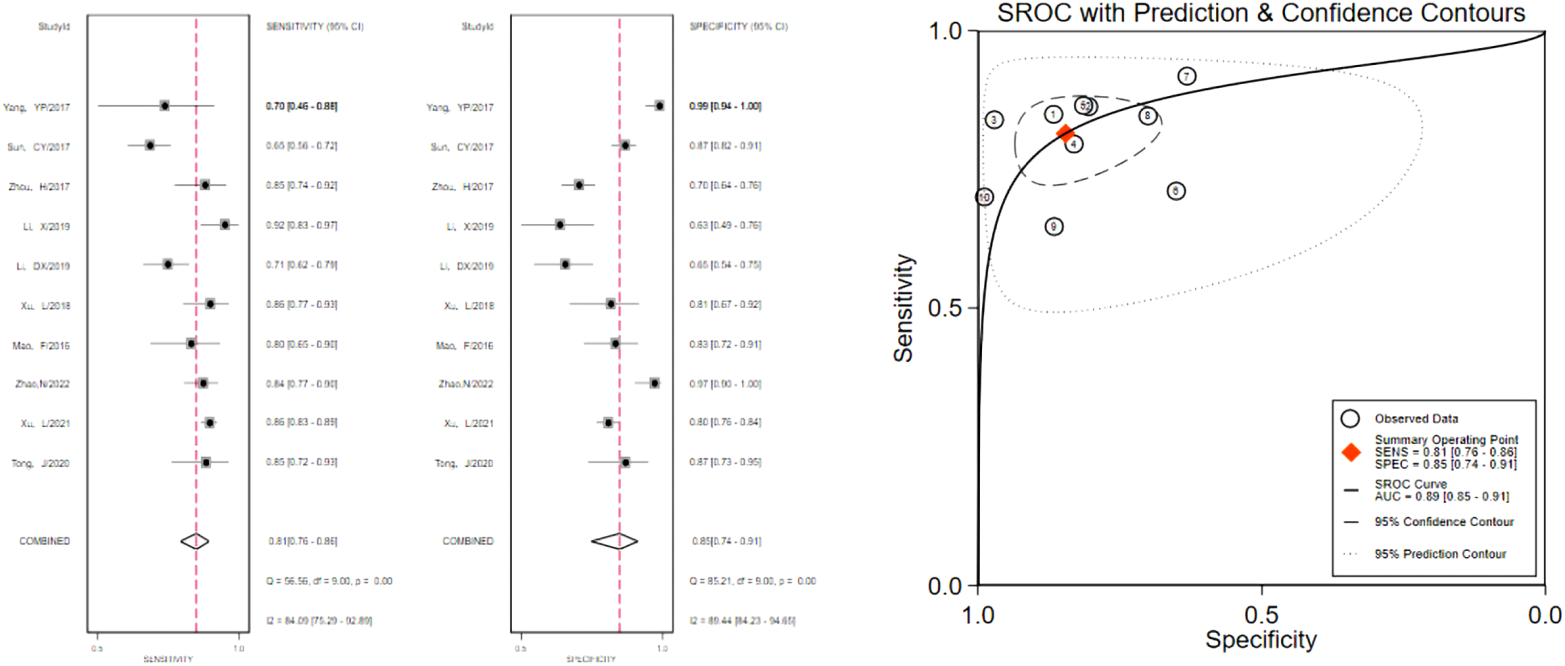
The sensitivity and specificity of the B_VTIQ_ group in the diagnosis of thyroid nodules and the summary ROC (Summary ROC) curve of the B_VTIQ_ group were analyzed. the AUC indicates the area under the curve.

The pooled 57 papers were divided into two groups according to the different pathological characteristics of malignant nodes, with group A being studies in which all included malignant nodes were papillary carcinomas, 21 in total, and group B being studies in which included malignant nodes included medullary carcinomas, follicular carcinomas, undifferentiated carcinomas, and other metastatic carcinomas in addition to papillary carcinomas, 36 in total (see **Tables 1** and **2** for the specific data of the two groups, respectively), and then classified according to VTQ and VTIQ two techniques were classified again, and groups A and B were divided into A_VTQ_ group, A_VTIQ_ group,B_VTQ_ group and B_VTIQ_ group, respectively. Regression analysis was done for each of the four data groups, and the data showed that the sensitivity, specificity, and AUC of the A_VTQ_ group were 0.82 (95CI%, 0.76-0.87), 0.84 (95CI%, 0.78-0.89), and 0.90 (95CI%, 0.87-0.92) (**Figure 2**), respectively; the sensitivity, specificity, and AUC of the A_VTIQ_ group were 0.75 (95CI%, 0.69-0.80), 0.83 (95CI%, 0.75-0.89) and 0.79 (95CI%, 0.75-0.82), respectively (**Figure 3**); the sensitivity, specificity and AUC of B_VTQ_ group were 0.82 (95CI%, 0.77-0.85), (95CI%, 0.82-0.90) and 0.90 (95CI%, 0.87-0.93) (**Figure 4**); the sensitivity, specificity, and AUC of the B_VTIQ_ group were 0.81 (95CI%, 0.76-0.86), 0.85 (95CI%, 0.74-0.91), and 0.89 (95CI%, 0.85-0.91), respectively (**Figure 5**). The data from the A_VTQ_ group were compared with the B_VTQ_ group and the A_VTIQ_ group with the B_VTIQ_ group. The results of data analysis showed that the difference in sensitivity and specificity between the A_VTQ_ and B_VTQ_ groups was small and not statistically significant (*p* < 0.01 for both sensitivity and specificity), and there was no difference in sensitivity but a difference in specificity between the A_VTIQ_ and B_VTIQ_ groups and the difference was statistically significant (*p* = 0.08 > 0.05).

**Table 1:** Characteristics of Group A literature.

Author	Study region	year	Number of nodules	Benign/Malignant	Malignant %	Sensitive	Specificity	TP	FP	FN	TN	Cut-off m/s	Type
Xiao,LL (14)	China	2012	67	28/39	58.2	76.9	78.6	30	6	9	22	2.78	VTQ
Hou,XJ (15)	China	2013	85	65/20	23.5	80.0	89.2	16	7	4	58	2.42	VTQ
Dong,FJ (16)	China	2015	55	28/27	49.1	88.9	96.4	24	1	3	27	2.42	VTQ
Liu,BJ (17)	China	2017	141	70/71	50.4	76.1	70.0	54	21	17	49	2.58	VTQ
Wu,L (18)	China	2018	88	56/32	36.4	78.1	91.1	25	5	7	51	2.80	VTQ
Chen,L (19)	China	2013	78	50/28	35.9	71.4	86.0	20	7	8	43	3.18	VTQ
Zhang,YF (20)	China	2014	173	77/96	55.5	56.2	79.2	54	16	42	61	3.10	VTQ
Huang,R (21)	China	2018	51	17/34	66.7	76.5	94.1	26	1	8	16	2.19	VTQ
Zhang,FJ (22)	China	2013	155	93/62	40.0	96.8	95.7	60	4	2	89	2.84	VTQ
Jiang,LY (23)	China	2016	195	103/92	47.2	72.8	77.7	67	23	25	80	2.98	VTQ
Sha,YM (24)	China	2017	95	24/71	74.7	88.7	95.8	63	1	8	23	2.67	VTQ
Song,HY (25)	China	2014	193	136/57	29.5	97.0	81.0	55	26	2	110	2.48	VTQ
Xing,P (26)	China	2016	90	54/36	40.0	80.6	74.1	29	14	7	40	2.57	VTQ
Ha,seung Mi (27)	Korea	2016	198	168/30	15.2	86.7	50.6	26	83	4	85	2.37	VTQ
Ke,K (28)	China	2017	69	37/32	46.4	87.5	86.5	28	5	4	32	2.55	VTQ
Zhang,YF (29)	China	2012	173	129/44	25.4	75.0	82.2	33	23	11	106	2.87	VTQ
Zhang,Y (30)	China	2019	62	22/40	64.5	77.5	63.6	31	8	9	14	3.00	VTIQ
Wu,SN (31)	China	2016	51	16/35	68.6	88.6	93.7	31	1	4	15	2.49	VTIQ
Li,DX (32)	China	2017	186	82/104	55.9	72.1	87.8	75	10	29	72	2.91	VTIQ
He,YP (33)	China	2017	75	49/26	34.7	65.4	83.7	17	8	9	41	3.51	VTIQ
Peng,QH (34)	China	2019	85	36/49	57.6	73.5	80.6	36	7	13	29	3.20	VTIQ

**Table 2:** Characteristics of Group B literature.

Author	Study region	year	Number of nodules	Benign/Malignant	Malignant %	Sensitive	Specificity	TP	FP	FN	TN	Cut-off m/s		Type
Cao,DM (35)	China	2019	148	120/28	18.9	81.2	64.7	23	42	5	78		3.30	VTQ
Deng,J (36)	China	2014	175	119/56	32.0	80.4	84.0	45	19	11	100		2.59	VTQ
Wang,R (37)	China	2015	129	70/59	45.7	67.8	91.4	40	6	19	64		2.43	VTQ
Ning,CP (38)	China	2014	179	64/115	64.2	74.8	73.4	86	17	29	47		2.47	VTQ
Zhou,J (1)	China	2014	191	122/69	36.1	96.3	96.2	66	5	3	117		2.55	VTQ
Gu,JY (39)	China	2012	98	76/22	22.4	86.4	93.4	19	5	3	71		2.56	VTQ
Wang,XY (40)	China	2016	88	59/29	33.0	75.9	94.9	22	3	7	56		2.57	VTQ
Hamidi,C (41)	Italian	2015	95	62/33	34.7	100.0	82.3	33	11	0	51		2.75	VTQ
Bojunga,J (42)	Germany	2012	158	137/21	13.3	57.0	85.0	12	20	9	117		2.57	VTQ
Zhan,J (43)	China	2015	170	102/68	40.0	79.4	84.3	54	16	14	86		2.75	VTQ
Chen,SH (44)	China	2014	125	62/63	50.4	75.0	70.0	47	19	16	43		2.50	VTQ
Hou,JX (45)	China	2014	44	19/25	56.8	88.0	94.7	22	1	3	18		2.76	VTQ
Zou,X (46)	China	2014	144	65/79	54.9	84.8	75.4	67	16	12	49		2.79	VTQ
Xu,JM (47)	China	2014	441	325/116	26.3	71.6	83.4	83	54	33	271		2.87	VTQ
Chen,Q (48)	China	2018	271	162/109	40.2	76.2	86.4	83	22	26	140		2.81	VTQ
Yang,YP (49)	China	2017	107	87/20	18.7	70.0	95.4	14	4	6	83		2.83	VTQ
Zhang,FJ (50)	China	2017	152	97/55	36.2	78.2	83.5	43	16	12	81		2.87	VTQ
Li,J (51)	China	2015	100	77/23	23.0	91.3	85.7	21	11	2	66		2.88	VTQ
Xu,JM (52)	China	2014	183	117/66	36.1	68.2	76.9	45	27	21	90		2.87	VTQ
Zhang,FJ (53)	China	2014	113	67/46	40.7	91.3	85.1	42	10	4	57		2.90	VTQ
Du,YR (54)	China	2018	142	70/72	50.7	91.7	60.0	66	28	6	42		2.31	VTQ
Zhang,HP (55)	China	2014	71	39/32	45.1	71.9	100.0	23	0	9	39		2.91	VTQ
Ni,JN (56)	China	2013	275	152/123	44.7	91.1	82.3	112	27	12	125		2.35	VTQ
Jung,WS (57)	Korea	2016	127	95/32	25.2	75.0	91.0	24	9	8	86		3.28	VTQ
Pandey,NN (58)	India	2017	40	26/14	35.0	85.7	96.2	12	1	2	25		2.53	VTQ
Grazhdani H (59)	Italy	2014	82	60/22	26.8	95.0	75.0	21	15	1	45		2.46	VTQ
Tong,J (60)	China	2020	98	45/53	54.1	84.9	86.7	45	6	8	39		2.96	VTIQ
Xu,L (61)	China	2020	922	405/517	56.1	86.3	80.5	446	79	71	326		3.55	VTIQ
Zhao,N (62)	China	2022	212	69/143	67.5	83.9	96.7	120	2	23	67		2.66	VTIQ
Mao,F (63)	China	2016	109	65/44	40.4	79.5	83.1	35	11	9	54		2.92	VTIQ
Xu,L (64)	China	2018	117	43/74	63.2	86.7	82.3	64	8	10	35		3.03	VTIQ
Li,DX (65)	China	2019	204	83/121	59.3	71.1	65.1	86	29	35	54		2.74	VTIQ
Li,X (66)	China	2019	130	57/73	56.2	92.3	63.2	67	21	6	36		2.80	VTIQ
Zhou,H (67)	China	2017	302	237/65	21.5	84.6	70.0	55	71	10	166		2.60	VTIQ
Sun,CY (68)	China	2017	388	238/150	38.7	64.7	86.6	97	32	53	206		3.15	VTIQ
Yang,YP (49)	China	2017	107	87/20	18.7	70.0	98.8	14	1	6	86		3.01	VTIQ

Then, the 58 papers were divided into two groups according to the two techniques of VTIQ and VTQ, and each group was sorted according to the size of the cut-off value from smallest to largest, and then the sensitivity, specificity and AUC of each group were calculated in every three groups. The sensitivity, specificity, and AUC of VTQ were 0.91 (95CI%, 0.80-0.97), 0.88 (95CI%, 0.73-0.95), and 0.96 (95CI%, 0.93-0.97), respectively; when the cut-off value was in the interval of 3.01-3.15m/s, the diagnostic efficacy of VTIQ was the best. The best diagnostic performance of VTIQ was achieved when the cut-off value was in the interval of 3.01-3.15m/s, with sensitivity, specificity and AUC of 0.74 (95CI%, 0.59-0.58), 0.92 (95CI%, 0.75-0.98) and 0.88 (95CI%, 0.84-0.90), respectively.

### Publication bias

**Figure 6:**
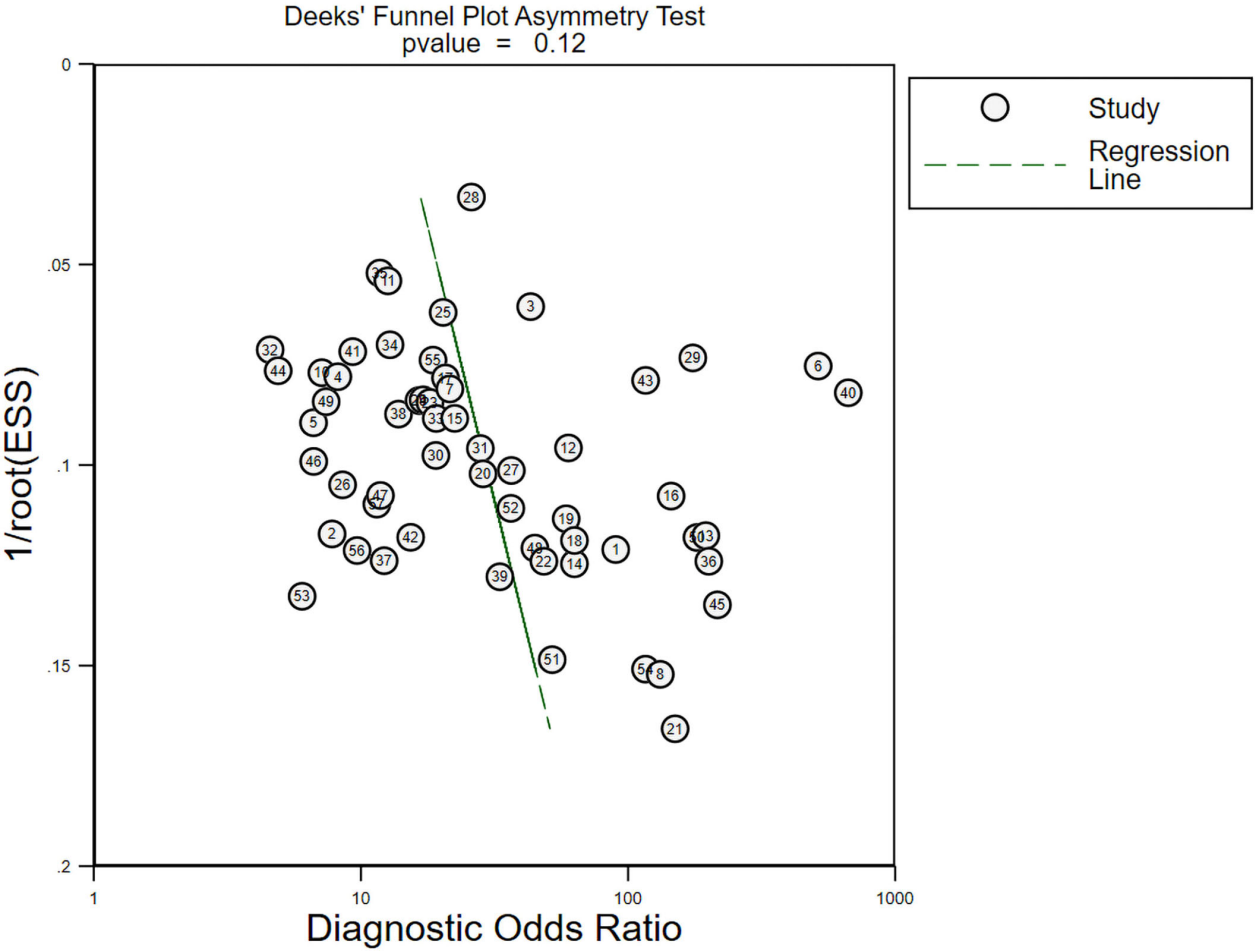
Publication bias assessment of all included literature.

When using meta-analysis in diagnostic trials, Deeks funnel plots are usually chosen to assess publication bias, and the results of Deeks funnel plots are shown in **Figure 6**. p > 0.05, suggesting no publication bias in this study.

### Literature quality assessment

**Figure 7:**
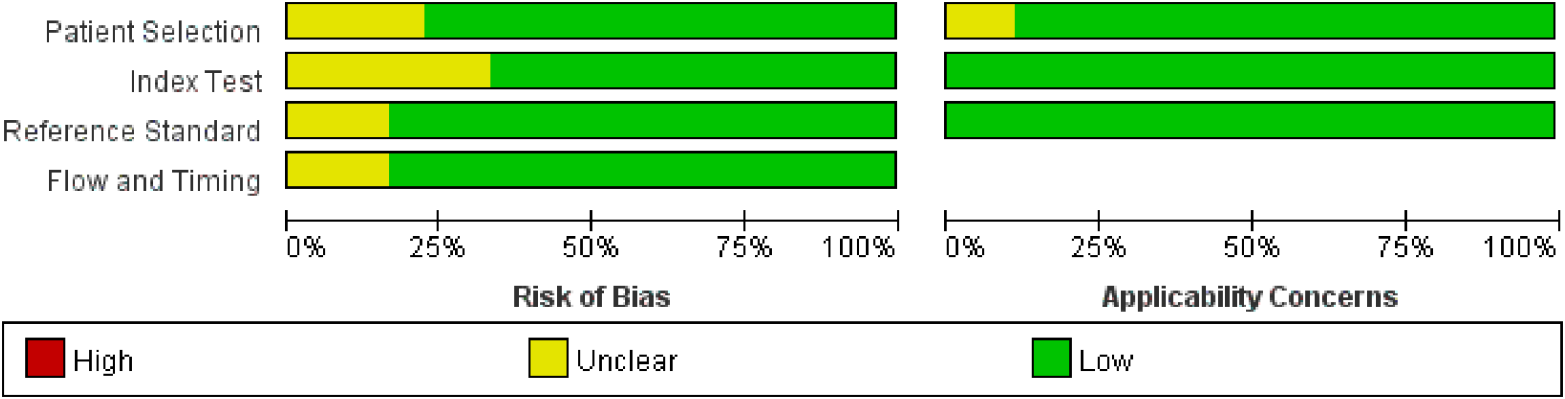
Literature quality evaluation of group A.

**Figure 8:**
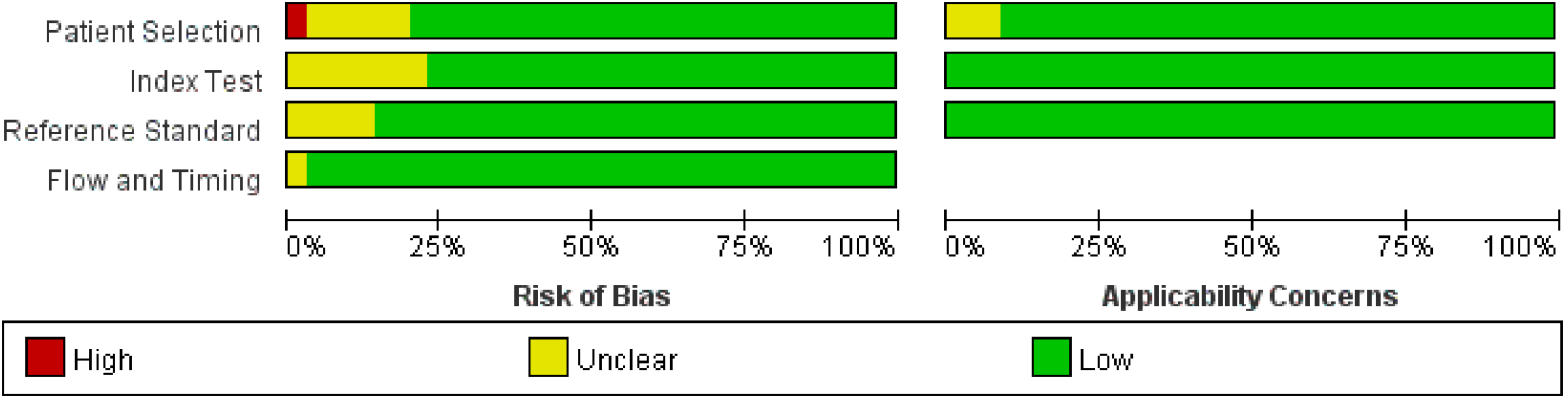
Literature quality evaluation of group B.

All included literature was evaluated for quality using RevMan 5.3, and the results of the literature quality evaluation are shown in **Figures 7** and **8**.

## Discussion

In this study, the included literature was divided into four groups according to whether all malignant nodules were papillary thyroid carcinomas and the difference between VTQ and VTIQ. From the results, it is clear that there was a statistically significant difference in specificity between group A and group B only when VTIQ was used to identify benign and malignant thyroid nodules, and the specificity of diagnosis was better when non-papillary thyroid carcinomas were included in malignant thyroid nodules.

From past studies, it is known that non-thyroidal papillary carcinomas such as follicular and medullary carcinomas are pathologically different from papillary thyroid carcinomas, with follicular and medullary carcinomas having less fibrous content and more cellular components compared to papillary carcinomas. Papillary carcinomas are often accompanied by sand-like calcification formation, so the pathological specimens of papillary carcinomas are harder, while follicular and medullary carcinomas are softer in texture (11). However, the results of this study showed that there was no difference in the diagnostic efficacy of VTQ for malignant nodules regardless of whether they contained non-papillary thyroid carcinoma, whereas the specificity of VTIQ was superior for the group of malignant nodules containing non-papillary thyroid carcinoma.

This result was unexpected, for which several speculations were made: one, it may be because some malignant nodules such as follicular carcinoma and medullary carcinoma have more distinct ultrasound features due to their poor differentiation. According to the latest European Thyroid Association guidelines, when a lesion has one of the above features of irregular shape, irregular border, microcalcifications and deep hypoechogenicity, the nodule may be malignant up to 26-87%. The more malignant features a tumor has, the highest its risk of malignancy. In a study by Zhao,J 2020, it was shown that some medullary carcinomas have more obvious malignant ultrasound features, specifically the irregular morphology of the tumor, poor demarcation with surrounding tissues, solid hypoechoic or very hypoechoic, and intra-nodular calcification (69); secondly, it is also possible that there are many microscopic thyroid papillary carcinomas among the papillary thyroid carcinomas, and The ROI range of ARFI is 6mm×5mm, which is not suitable for the diagnosis of smaller nodules, and this may also be the reason for this result (70). For example, in the included study by Chen, SH in 2014, they included a total of 275 nodules and 23 microscopic papillary thyroid carcinomas out of 60 papillary thyroid carcinomas. The sensitivity of VTQ for thyroid nodules in that article was 75% and the specificity was 70% (44); in addition, all nodules included in Zhu,J’s article in 2015 were microscopic nodules, and the sensitivity of VTQ for diagnosing benign and malignant thyroid nodules in that study was 76.4% and the specificity was 75.8% (70).

In addition, the measurement range of SWV is 0.5-8.4 m/s, a characteristic that makes ARFI unable to achieve satisfactory measurement results for extremely hard or soft tissues, and the quality of imaging is difficult to guarantee (2). Therefore, it will have an unavoidable impact on the quality of the article. In addition to this, there are some studies that did not exclude nodules with a background of diffuse thyroid lesions when they were included. Pathologically, diffuse thyroid lesions are caused by infiltration of thyroid follicular cells by diffuse lymphocytes, destruction of follicles by atrophy and fibrosis, and these factors can make the texture of the thyroid gland harder (71). It is also possible that although the malignant nodules in group B included nonpapillary carcinomas, the proportion of nonpapillary carcinomas in thyroid cancer was so low that the results in this section were biased, but because of the low incidence of nonpapillary thyroid carcinomas and the paucity of data, there is not a large body of data to support the exclusion of this speculation.

The sensitivity, specificity and AUC of each group were calculated by comparing the data of each group and found that the best diagnostic efficacy was achieved when the cut-off values were in the range of 2.48-2.55m/s. The sensitivity, specificity and AUC of VTQ were 0.91 (95CI%, 0.80-0.97), 0.88 (95CI%, 0.73-0.95) and 0.96 (95CI%, 0.93-0.97), respectively, and the diagnostic efficacy of VTIQ was best when the cut-off value was in the range of 3.01-3.15m/s. The sensitivity, specificity and AUC of VTQ were best when the cut-off value was in the range of 3.01-3.15m/s. The diagnostic efficacy of VTIQ was best when the cut-off value was in the range of 3.01-3.15m/s, with sensitivity, specificity and AUC of 0.74 (95CI%, 0.59-0.58), 0.92 (95CI%, 0.75-0.98) and 0.88 (95CI%, 0.84-0.90), respectively.

### Limitation

This meta-analysis has several limitations. We searched only three databases, PubMed, Web of science, and Embase, suggesting that there may be relevant studies that were missed. Also, this meta-analysis included only English-language literature, so there may be language bias.

## Conclusion

In summary, ARFI imaging is a highly effective imaging tool to identify benign and malignant thyroid nodules. There is no difference in the diagnostic effectiveness of VTQ for malignant nodules with or without non-papillary thyroid cancer, while VTIQ has a better specificity for the diagnosis of malignant nodules with non-papillary thyroid cancer. Therefore, ARFI imaging of benign and malignant thyroid nodules must take into account various clinical information of the patient and be analyzed critically to make a more accurate diagnosis.

## Author contributions

JL, Y-RZ and X-WC contributed to the conception and design of the study. Y-RZ and P-SZ searched and reviewed studies, extracted and analyzed the data, and wrote the first draft of the manuscript. Q-LL, MC and J-YR reviewed and edited the manuscript. X-YG and T-TD directed the project and contributed to discussion as well as reviewed and edited the manuscript. All authors contributed to the article and approved the submitted version.
