# Corrigendum: Association between diagnostic efficacy of acoustic radiation force impulse for benign and malignant thyroid nodules and the presence or absence of non-papillary thyroid cancer: A meta-analysis

**DOI:** 10.3389/fonc.2023.1165490

**Published:** 2023-03-14

**Authors:** Jun Li, Yu-Rui Zhang, Jia-Yu Ren, Qiao-Li Li, Pei-Shan Zhu, Ting-Ting Du, Xiao-Yan Ge, Ming Chen, Xin Wu Cui

**Affiliations:** ^1^ Department of Ultrasound, The First Affiliated Hospital of Medical College, Shihezi University, Shihezi, China; ^2^ NHC Key Laboratory of Prevention and Treatment of Central Asia High Incidence Diseases, First Affiliated Hospital, School of Medicine, Shihezi University, Shihezi, China; ^3^ Department of Medical Ultrasound, Tongji Hospital, Tongji Medical College, Huazhong University of Science and Technology, Wuhan, China

**Keywords:** non-papillary thyroid cancer (NPTC), virtual touch quantification (VTQ), virtual touch tissue imaging and quantification (VTIQ), shear wave velocity (SWV), meta-analysis

In the published article, there was an error regarding the affiliation for Jun Li. As well as having affiliation 1, they should also have *NHC Key Laboratory of Prevention and Treatment of Central Asia High Incidence Diseases, First Affiliated Hospital, School of Medicine, Shihezi University, Shihezi, China*.

The authors apologize for this error and state that this does not change the scientific conclusions of the article in any way. The original article has been updated.

